# Surgical Resection of Abdominal Solid Organ Tumors With Inferior Vena Cava Extension: A Single-Center Experience With Long-Term Follow-Up

**DOI:** 10.7759/cureus.103335

**Published:** 2026-02-10

**Authors:** Nikoleta Stanitsa, Filippos-Paschalis Rorris, Konstantinos Antonopoulos, Emmanouil Tempelis, Aikaterini Dedeilia, Ilias Samiotis, Anastasios Athanasopoulos, Eleni Papagianni, Nikolaos Schizas, Panagiotis Dedeilias

**Affiliations:** 1 Cardiothoracic Surgery, Evangelismos General Hospital, Athens, GRC; 2 Cardiac Surgery, Onassis Hospital, Athens, GRC; 3 Vascular Surgery, "Attikon" University Hospital, School of Medicine, National and Kapodistrian University of Athens, Athens, GRC; 4 Cardiac Surgery, Evangelismos General Hospital, Athens, GRC; 5 Surgery, Boston Medical Center, Boston, USA; 6 Pathology, Evangelismos General Hospital, Athens, GRC; 7 Cardiac Surgery, "Attikon" University Hospital, School of Medicine, National and Kapodistrian University of Athens, Athens, GRC

**Keywords:** adrenocortical carcinoma, cardiopulmonary bypass, hypothermic circulatory arrest, inferior vena cava, long-term survival, radical resection, renal cell carcinoma, thrombectomy, transesophageal echocardiography, tumor thrombus

## Abstract

Introduction: Abdominal solid organ malignancies, particularly renal and adrenal tumors, may extend into the inferior vena cava (IVC) as tumor thrombus and, in rare cases, reach the right atrium. Surgical management is technically demanding and associated with significant perioperative risk. This study evaluates perioperative outcomes and long-term survival after radical resection of abdominal tumors with IVC extension using cardiopulmonary bypass (CPB).

Materials and methods: A prospectively maintained dataset was retrospectively analyzed for adult patients who underwent surgical management of abdominal solid organ tumors with IVC extension between June 2002 and September 2023 at a tertiary referral center. Procedures were performed by a multidisciplinary team. CPB was used in all cases. The extent of venous involvement was categorized using the Mayo Clinic classification system. The primary outcome was long-term survival, and secondary outcomes included perioperative complications and in-hospital mortality.

Results: Thirty-nine patients underwent resection (mean age 61 ± 11.3 years; 74.3% male). Tumors originated most commonly from the kidney (87.1%), followed by the adrenal gland (10.3%), with one case of primary IVC intimal sarcoma. Clear cell RCC was the predominant histological subtype. Mean operative duration was 390 minutes; mean CPB time was 145 minutes; mean ischemia time was 55 minutes; and mean hypothermic circulatory arrest (HCA) time was 33 minutes. In-hospital mortality was 10.3%, including two intraoperative deaths. Mean follow-up was 4.8 ± 3.5 years. Estimated overall survival at 1, 5, and 10 years was 87.1%, 64.1%, and 46.1%, respectively.

Conclusions: Radical resection of abdominal solid organ tumors with IVC extension using CPB is feasible and can provide acceptable long-term survival despite substantial perioperative risk. A multidisciplinary approach is essential to optimize outcomes in these complex cases.

## Introduction

Abdominal solid organ malignancies with venous tumor thrombus extension into the inferior vena cava (IVC) represent one of the most challenging scenarios in contemporary oncologic surgery. Renal cell carcinoma (RCC) is the most common malignancy associated with venous invasion, occurring in up to 10% of cases, while supradiaphragmatic or intracardiac extension is observed in approximately 1% of patients [1,2]. Less frequently, adrenocortical carcinoma (ACC), hepatocellular carcinoma (HCC), and rare primary vascular tumors such as intimal sarcoma may also demonstrate aggressive intravascular growth patterns [3]. These tumors are often biologically advanced and require meticulous surgical planning to achieve oncologic control while minimizing perioperative risk.

The anatomic extent of tumor thrombus strongly influences both clinical presentation and operative strategy. Lower-level IVC involvement may be asymptomatic or present with nonspecific findings, whereas higher-level extension can result in significant hemodynamic compromise, including lower-extremity edema, hepatic congestion, pulmonary embolization, and acute right-sided heart failure in cases of intracardiac extension. The Mayo Clinic classification system has become the most widely adopted framework for describing the cranial extent of tumor thrombus and plays a central role in operative planning and interdisciplinary communication [4]. Increasing thrombus level is associated with greater technical complexity, prolonged operative times, and higher perioperative morbidity and mortality [5].

Complete surgical resection with tumor thrombectomy remains the only potentially curative treatment for patients with non-hematologic malignancies and IVC tumor thrombus. Operative strategies range from caval control without extracorporeal circulation to more complex approaches incorporating venovenous bypass or cardiopulmonary bypass (CPB), often with hypothermic circulatory arrest (HCA) for extensive level III-IV thrombi [6]. Liver transplantation-derived “piggyback” techniques have also been described as alternatives to CPB and may be effective in carefully selected patients [7,8]. However, these non-CPB approaches may be limited in cases of dense caval wall invasion, supradiaphragmatic or intracardiac extension, or hemodynamic instability during caval manipulation [9].

The use of CPB with HCA provides several advantages in advanced cases, including controlled hemodynamics, a bloodless operative field, and excellent exposure of the supradiaphragmatic IVC and right atrium. Although concerns have historically been raised regarding coagulopathy and neurologic complications, recent evidence suggests that CPB is not an independent predictor of adverse outcomes when used in appropriately selected patients at experienced centers [10,11]. Outcomes are strongly influenced by institutional expertise, surgical volume, and the availability of a coordinated multidisciplinary team [12].

Despite multiple published series, most available data derive from single-center experiences with heterogeneous patient populations and limited long-term follow-up. Reporting institutional outcomes with consistent operative principles therefore remains valuable. In the present study, we report our single-center experience with radical resection of abdominal solid organ tumors with IVC extension using CPB and HCA, focusing on perioperative outcomes and long-term survival.

## Materials and methods

A prospectively maintained dataset was compiled for all adult patients who underwent surgical management of solid abdominal tumors with extension into the IVC at a single tertiary referral center between June 2002 and September 2023. Approval for data usage was obtained from the Institutional Review Board of Evangelismos General Hospital, and the requirement for informed consent was waived due to the retrospective nature of the analysis. All medical records were retrospectively reviewed and fully de-identified prior to analysis in accordance with institutional and ethical guidelines.

Detailed operative and postoperative characteristics were available for all included patients. The primary outcome was long-term survival. Secondary outcomes included perioperative complications and in-hospital mortality. Patients with hematologic malignancies or incomplete operative data were excluded. The extent of tumor thrombus involvement in the IVC was categorized using the Mayo Clinic classification system.

Statistical analysis

Continuous variables are presented as mean ± standard deviation (SD) and range. Categorical variables are presented as absolute numbers and percentages. Overall survival was defined as the time from the date of surgery to death from any cause or last follow-up. Survival probabilities were estimated using the Kaplan-Meier method, with censoring at the last known follow-up. Statistical analyses were descriptive given the sample size.

Surgical technique

All procedures were conducted by a multidisciplinary team, including cardiothoracic surgeons and either urologists or general surgeons, depending on the tumor’s primary site. CPB was employed in all cases to provide circulatory support and facilitate complete tumor resection. CPB configurations varied according to surgeon preference while adhering to the same operative principles.

Following an initial laparotomy and tumor mobilization by the abdominal team, a median sternotomy was performed to enable cardiothoracic access. Systemic heparinization was administered, and CPB was established under systemic hypothermia. All patients underwent HCA. Arterial cannulation was performed via the ascending aorta in all cases, except one in which the subclavian artery was used due to anatomical constraints. Venous cannulation was achieved via the superior vena cava and the femoral vein contralateral to the tumor using a short venous cannula.

**Figure 1:**
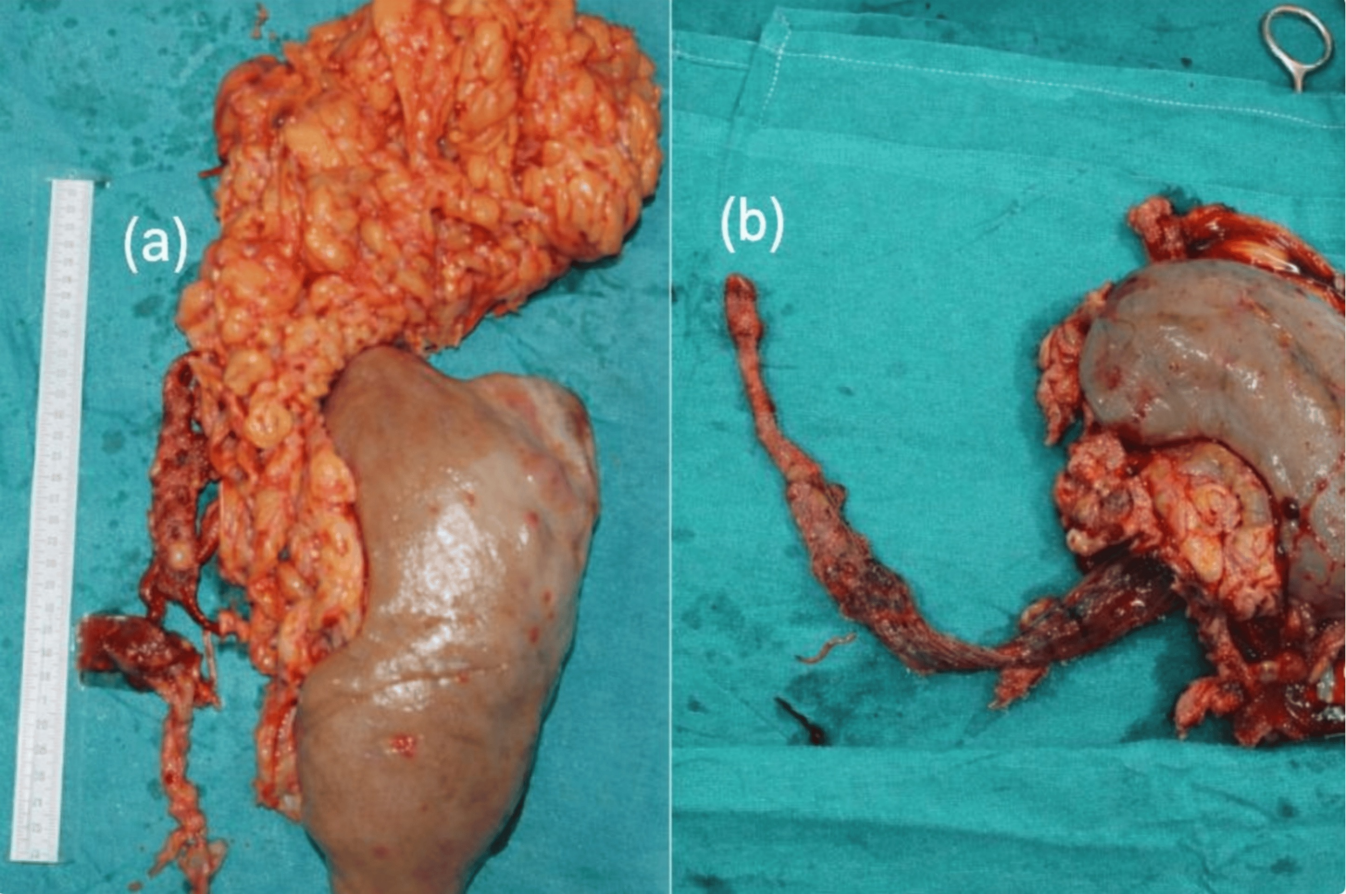
Inferior vena cava tumor thrombus resection (a) Intraoperative exposure and longitudinal incision of the inferior vena cava (IVC), providing controlled access to the venous lumen for tumor thrombus removal. (b) En bloc extraction of the tumor thrombus from the IVC following venotomy, demonstrating complete removal of the intraluminal tumor component to achieve vascular clearance.

**Figure 2:**
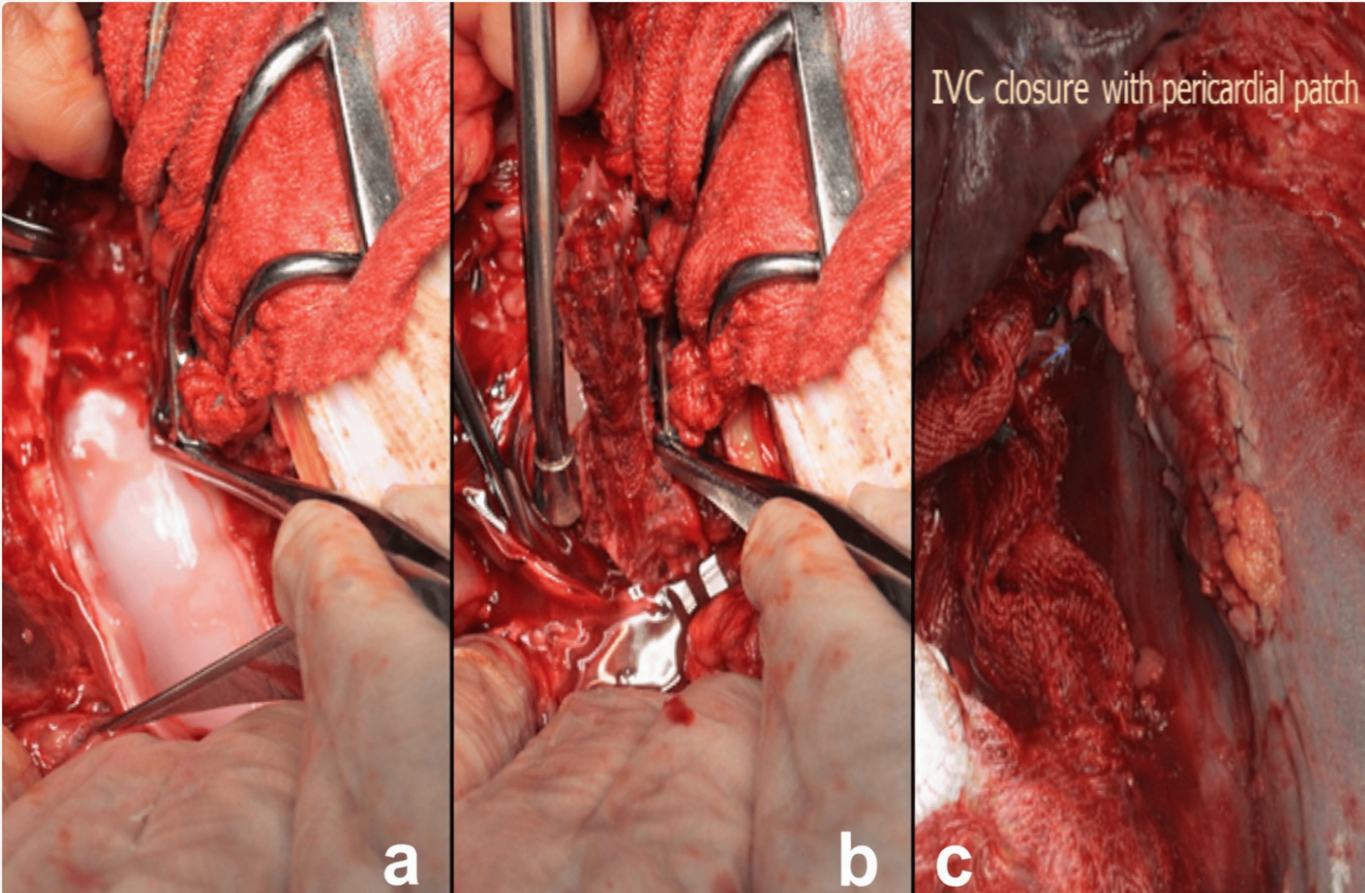
En bloc resection of renal tumor with inferior vena cava extension (a) Intraoperative view demonstrating surgical exposure of the inferior vena cava (IVC) following venotomy during tumor thrombus resection. The venous lumen is clearly visualized, and a measurement scale is included to illustrate the size and extent of the resected specimen. (b) Intraoperative image showing en bloc extraction of the tumor thrombus from the IVC, highlighting complete removal of the intraluminal tumor component together with the primary renal specimen and surrounding perirenal tissue. (c) Closure of the IVC using a pericardial patch. The image demonstrates the surgical repair of the IVC following tumor thrombus removal, ensuring vascular continuity and preventing stenosis. The pericardial patch is sutured securely in place to restore vessel integrity.

After cooling to the target core temperature, HCA was initiated, and the IVC was opened distal to the tumor origin (Figure 1a). The IVC and adjacent structures were inspected to assess wall involvement and the feasibility of en bloc resection. En bloc resection included the affected organ, involved vascular structures, and adherent tissues (Figures 2a, 2b). For thrombus extending into the supradiaphragmatic IVC and/or right atrium, thrombus extraction was performed via right atriotomy when necessary (Figure 1b). Additional intraluminal thrombi were extracted, and the IVC was reconstructed using primary closure or patch angioplasty, depending on the extent of resection. Patch angioplasty with bovine or autologous pericardium was performed in 15.4% (n = 6) of patients (Figure 2c).

Following tumor removal, CPB was reinstituted, and systemic rewarming was initiated. Separation from CPB was guided by intraoperative transesophageal echocardiography (TEE) with real-time assessment. Abdominal lymphadenectomy was performed as indicated by tumor staging.

Mean total operative duration was 390 minutes (range: 200-640 minutes), mean CPB time was 145 minutes (range: 95-217 minutes), mean ischemia time was 55 minutes (range: 32-137 minutes), and mean HCA time was 33 minutes (range: 16-126 minutes) for the entire cohort. Postoperatively, patients were transferred to the cardiac surgery intensive care unit (cICU) for close monitoring and recovery.

## Results

During the study period, 39 patients who underwent abdominal solid organ tumor resection with IVC extension were identified. The mean patient age was 61 ± 11.3 years, and 74.3% (n = 29) were male. Baseline patient and tumor characteristics are summarized in Table 1. Mean follow-up duration was 4.8 ± 3.5 years. At the last follow-up, 46.2% of patients (n = 18) remained alive.

**Table 1:** Patient characteristics

Variable	N (%)
Age	61 ± 11.3 years
Male gender	29(74.3%)
Affected organ	
Kidney	34 (87.1%)
Adrenal gland	4 (10.3%)
IVC	1 (2.5%)
Histology	
Clear cell RCC	34 (87.1%)
Adrenocortical carcinoma	3 (7.6%)
HCC of adrenal gland	1 (2.5%)
Intimal sarcoma	1 (2.5%)
TNM stage (kidney)	
T2bN0M0	2
T3aN0M0	2
T3bN0M0	10
T3cN0M0	8
T3cN1M0	4
T4N0M0	2
T4N1M0	5
T4N0M1	1
TNM stage (adrenal gland primary tumors)	
T3N0M1	1
T4N0M0	1
T4N0M1	1
Mayo Clinic classification	
II	24
III	7
IV	8

Tumor origin and laterality

The majority of tumors originated from either the kidney or adrenal gland (97.4%; n = 38). Kidney tumors accounted for 87.1% (n = 34), adrenal tumors accounted for 10.3% (n = 4), and one patient had a primary IVC tumor (2.6%; n = 1). The right kidney was the most commonly affected site, accounting for 74.3% (n = 29) of cases. All adrenal primary tumors were right-sided (n = 4).

Metastatic presentation and urgent surgery

Four patients presented with distant metastatic disease at the time of surgery. These patients underwent urgent operations without preoperative oncologic consultation due to acute heart failure symptoms and resultant cardiogenic shock, attributed to diminished preload caused by right atrial occupancy by the tumor thrombus.

Histology and pathologic characteristics

Clear cell RCC was the most common diagnosis (87.1%; n = 34). ACC was diagnosed in 7.7% (n = 3). Rare diagnoses included an ectopic HCC of the adrenal gland (2.6%; n = 1) and primary IVC intimal sarcoma (2.6%; n = 1) (Table 1).

**Figure 3:**
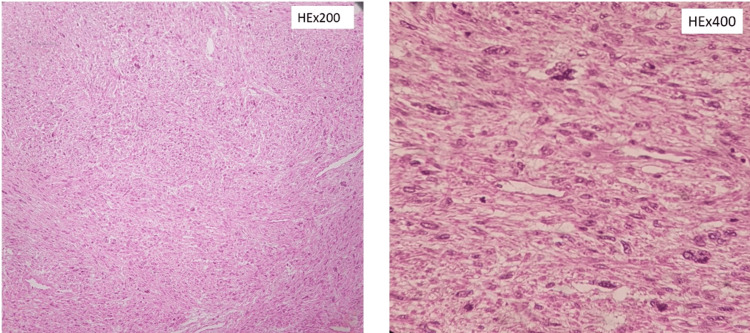
Histopathological features of inferior vena cava intimal sarcoma Tumor composed of epithelioid, pleomorphic, and spindle cells with high-grade atypia, arranged in fascicles.

**Figure 4:**
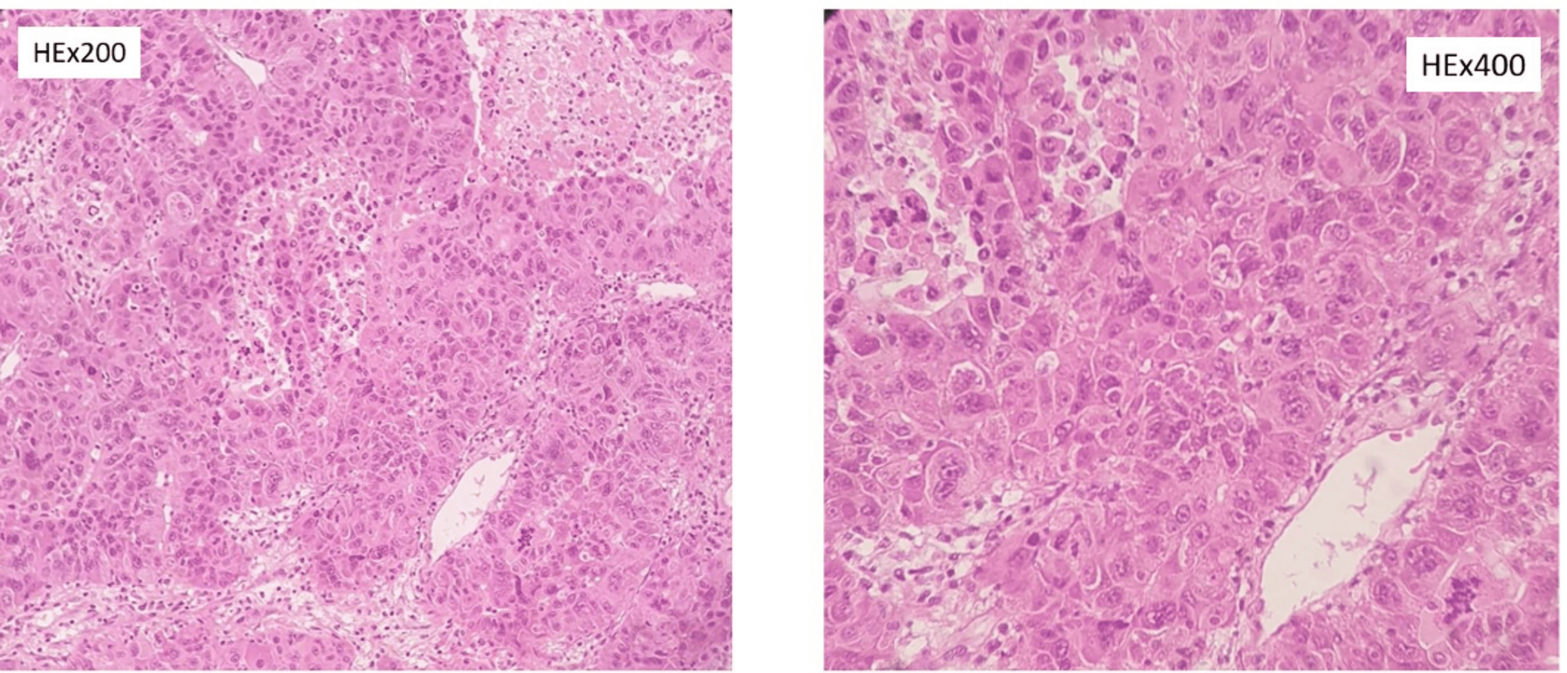
Ectopic hepatocellular carcinoma of the adrenal gland High-grade carcinoma composed of neoplastic cells with hepatoid-like features (hepatoid differentiation). Necrosis and high mitotic activity are present.

**Figure 5:**
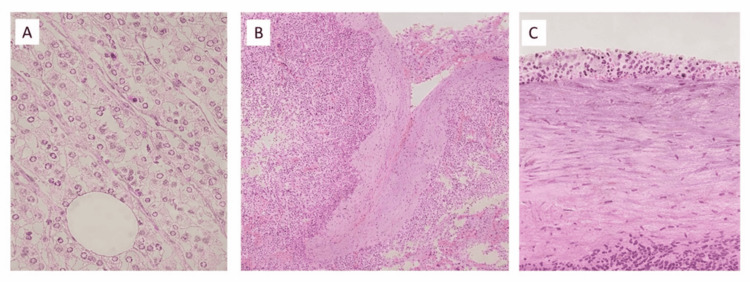
Adrenocortical carcinoma invading the inferior vena cava (A) Adrenal cortical neoplastic cells with moderate atypia and high mitotic activity (HE x400). (B, C) Invasion of the inferior vena cava by adrenocortical neoplastic cells.

In renal tumors, pathological staging most commonly corresponded to T3 disease. One RCC patient had pulmonary tumor thrombus embolism and was classified as M1 disease (2.9%; n = 1) within the renal subgroup. In the adrenal subgroup, three out of four adrenal tumors were adrenocortical carcinoma; among these, 100% (n = 3) were classified as M1 according to the provided pTNM distribution for ACC in Table 1. Tumor extension into the IVC and right atrium is considered stage IV disease for ACC according to ENSAT. Representative histologic images are shown in Figures 3-5.

Mayo Clinic classification

Tumor thrombus extent by Mayo Clinic classification was as follows: level II, 61.5% (n = 24); level III, 17.9% (n = 7); and level IV, 20.5% (n = 8) (Table 1).

In-hospital mortality and morbidity

Overall, in-hospital mortality was 10.3% (n = 4). Intraoperative death occurred in 5.1% (n = 2), attributed to low cardiac output syndrome or uncontrollable bleeding. Postoperative mortality occurred in 5.1% (n = 2), due to multi-organ failure (2.6%; n = 1) and sepsis (2.6%; n = 1) (Table 2). Mean length of stay in the cICU was 4.1 ± 11.2 days, and mean ward length of stay was 11.6 ± 5.7 days.

**Table 2:** Causes of death

Cause of death	Number of events (%)
Early in-hospital	4 (10.3%)
Intraoperative	2 (5.1%)
Multi-organ failure	1 (2.6%)
Sepsis	1 (2.6%)
Long term	17 (43.6%)
Sudden cardiac death	13 (33.3%)
Fatal stroke	2 (5.1%)
Contralateral renal tumor	1 (2.6%)
Metastatic disease	1 (2.6%)

Long-term follow-up

**Figure 6:**
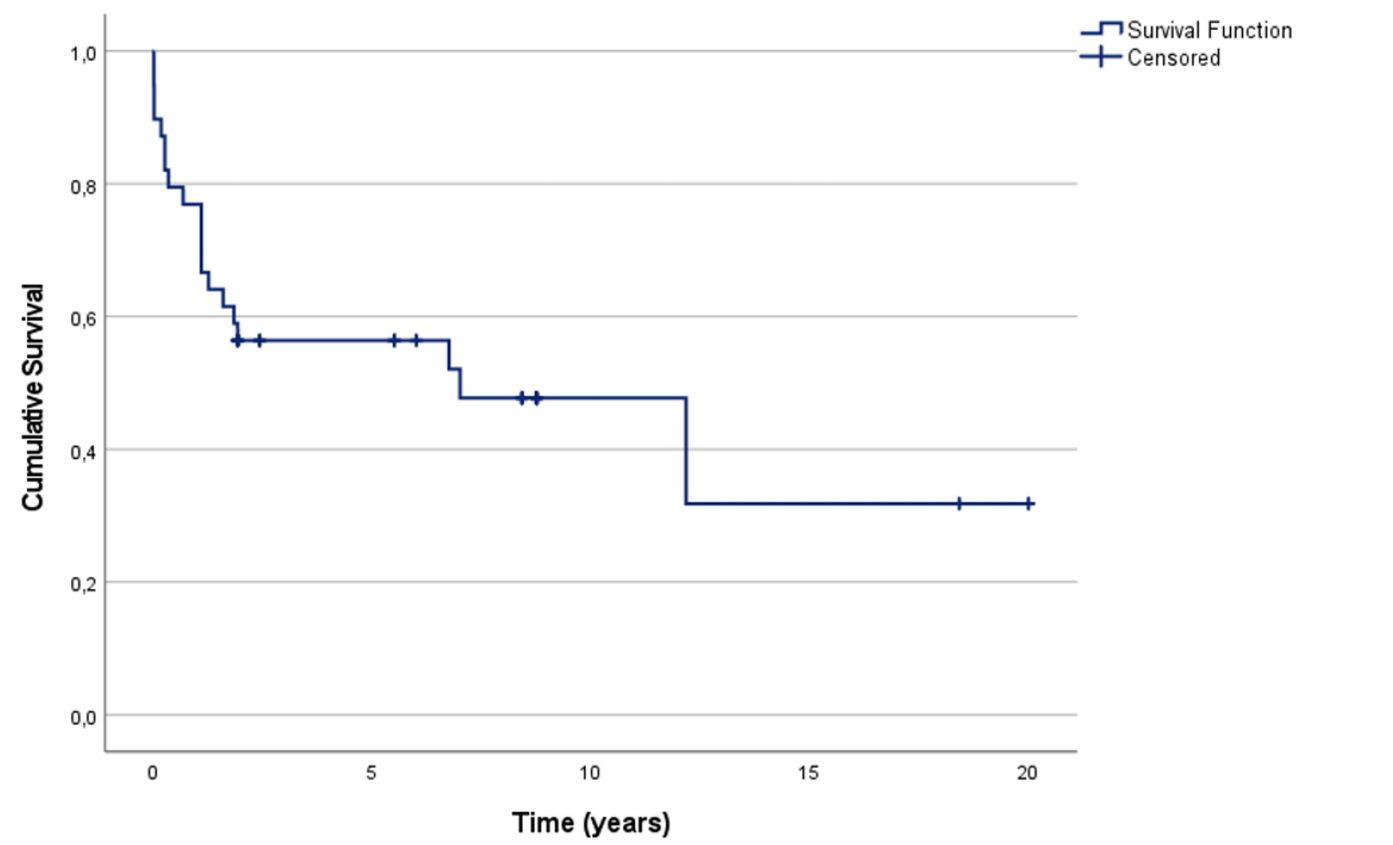
Kaplan-Meier survival curve Kaplan-Meier curve for overall survival (OS) after resection of abdominal solid organ tumors with inferior vena cava (IVC) tumor thrombus.

Follow-up data were available for up to 20 years after surgery. Kaplan-Meier-estimated overall survival at 1, 5, and 10 years was 87.1% (n = 34), 64.1% (n = 25), and 46.1% (n = 18), respectively (Figure 6). Sudden cardiac death was the most frequently recorded cause of late mortality (33.3%; n = 13) (Table 2). One patient developed a contralateral renal tumor during follow-up (2.6%; n = 1). Stroke occurred in 10.3% (n = 4) at a mean of 2.3 years postoperatively; fatal stroke accounted for 5.1% (n = 2) of late deaths (Table 2).

## Discussion

In the present study, we describe our surgical technique and institutional experience in the management of abdominal solid organ tumors with IVC extension using a standardized multidisciplinary approach. These cases represent some of the most technically demanding procedures in contemporary oncologic surgery and require meticulous preoperative planning and close intraoperative collaboration between cardiothoracic surgeons, vascular surgeons, urologists or general surgeons, anesthesiologists, and perfusionists [1,2]. The complexity of these operations arises not only from the need to achieve oncologically complete resection but also from the requirement to maintain hemodynamic stability while addressing extensive venous and, in some cases, intracardiac tumor involvement.

Tumor histology and pathologic heterogeneity

Although clear cell RCC constituted the majority of tumors in our cohort, we also encountered uncommon neoplastic entities, including ACC and primary intimal sarcoma, capable of aggressive intravascular growth and IVC invasion. The presence of these rare tumor types underscores the heterogeneity of abdominal solid organ malignancies associated with venous tumor thrombus and highlights the importance of individualized surgical planning based on both anatomic and histopathologic considerations [3].

Perioperative mortality and safety

Perioperative mortality remains a major concern in patients undergoing resection of tumors with extensive IVC involvement. In our series, in-hospital mortality occurred in 4/39 patients (10.3%), a rate comparable to those reported by contemporary high-volume centers, where mortality for Mayo level III-IV tumor thrombi typically ranges from 5% to 15% [4,5]. In addition, registry-based analyses have demonstrated a strong volume-outcome relationship in this pathology, with higher institutional experience associated with improved perioperative outcomes and reduced failure-to-rescue rates [6].

Consistent with prior reports, mortality in our cohort was higher among patients with advanced tumor thrombus levels, reflecting longer operative times, the need for intracardiac tumor extraction, and complex venous reconstruction [4].

Surgical strategy and the role of cardiopulmonary bypass

The optimal surgical strategy for managing IVC tumor thrombus remains a subject of debate. Alternative approaches, including liver transplantation-derived “piggyback” techniques, aim to avoid the systemic inflammatory response and coagulopathy associated with CPB [7,8]. However, these non-CPB techniques may be technically limited in patients with extensive supradiaphragmatic or intracardiac thrombus, dense caval wall invasion, or significant hemodynamic instability during caval manipulation [9].

In our practice, CPB with HCA was employed in all patients. Recent evidence supports this approach in advanced tumor thrombus. Tobert et al. demonstrated that CPB was not an independent predictor of increased perioperative complications or worse overall survival when tumor complexity and thrombus extent were taken into account [10]. Furthermore, a systematic review and meta-analysis comparing bypass versus non-bypass surgery for tumor thrombus extending into the IVC or right atrium concluded that CPB provides superior operative exposure and may reduce the risk of intraoperative tumor embolization in complex cases [11].

Oncologic outcomes and prognosis

Kaplan-Meier-estimated overall survival in our cohort was 64.1% at 5 years and 46.1% at 10 years, closely paralleling outcomes reported in other long-term institutional series [4,12]. While higher thrombus levels were historically considered a marker of poor prognosis, emerging evidence suggests that when complete (R0) resection is achieved, thrombus extent alone may not independently predict long-term survival. Instead, tumor biology, histologic subtype, and the presence of true distant metastatic disease appear to be the primary determinants of oncologic outcomes [13,14].

Histological challenges: ACC and primary vascular tumors

ACC with venous or atrial involvement is classified as stage IV disease according to the ENSAT due to its aggressive biological behavior [15]. Although complete surgical resection remains the cornerstone of potentially curative treatment, recurrence rates remain high, and adjuvant therapies such as mitotane may be required based on individual risk stratification [16]. Similarly, primary intimal sarcomas of the IVC are rare but highly aggressive malignancies, for which aggressive en bloc resection with negative margins remains the most important prognostic factor [17].

Emerging technologies

Minimally invasive approaches, including laparoscopic and robotic-assisted techniques, have been described for selected patients with lower-level tumor thrombi, demonstrating reduced blood loss and shorter hospital stays in carefully selected cases [18,19]. Nevertheless, for patients with Mayo level III-IV thrombi requiring extensive caval reconstruction and/or atrial exploration, open surgery with CPB remains the most reliable and oncologically sound strategy [20].

Neurologic morbidity

Neurologic morbidity remains an important consideration when using hypothermic circulatory arrest. In our cohort, stroke occurred in 4/39 patients (10.3%). The mean circulatory arrest time was 33 minutes, which falls within accepted safe limits for deep hypothermia. These findings suggest that patient-related factors, such as advanced age and pre-existing cerebrovascular disease, may contribute more significantly to neurologic outcomes than arrest duration alone [21].

Future directions

The postoperative management of high-risk RCC has evolved substantially during the latter part of the study period. The introduction of adjuvant immunotherapy, such as pembrolizumab, highlights the importance of close multidisciplinary collaboration following surgery and may further improve recurrence-free and overall survival in future cohorts [22].

## Conclusions

Radical surgical resection of abdominal solid organ tumors with IVC extension remains one of the most challenging procedures in oncologic surgery. In this single-center experience, the use of CPB and HCA enabled safe vascular control and complete tumor removal in a cohort characterized by advanced venous involvement, including a substantial proportion of Mayo level III and IV tumor thrombi. Despite substantial operative complexity and perioperative risk, acceptable early mortality and meaningful long-term survival were achieved. Our findings support the role of aggressive surgical management as the cornerstone of treatment for selected patients with non-hematologic abdominal malignancies and IVC tumor thrombus. Long-term survival rates observed in this study are comparable to those reported by other high-volume centers, reinforcing the potential benefit of complete resection even in cases with supradiaphragmatic or intracardiac extension. The presence of advanced venous tumor thrombus alone should therefore not be considered an absolute contraindication to surgery when complete resection is technically feasible.

Successful outcomes in these complex cases depend heavily on meticulous preoperative evaluation, careful patient selection, and close collaboration within a multidisciplinary team experienced in advanced oncologic, vascular, and cardiothoracic procedures. While CPB with HCA may not be necessary in all patients with IVC involvement, it represents a valuable and reproducible strategy for managing extensive thrombus and ensuring optimal operative exposure and hemodynamic stability. Further multicenter studies with larger cohorts and standardized reporting are warranted to better define optimal operative strategies, clarify prognostic factors, and evaluate the impact of adjuvant oncologic therapies. Nevertheless, the present study provides meaningful long-term data and supports the feasible continued use of aggressive, multidisciplinary surgical approaches for abdominal solid organ tumors with IVC extension.
